# Two Lectures on Water, Delivered to His Class, at Lebanon, Tennessee, by F. H. Gordon, M. D.

**Published:** 1850-08

**Authors:** 


### ECLECTIC DEPARTMENT,

### AND SPIRIT OF THE MEDICAL PERIODICAL PRESS.

From an article in the Western Journal of Medicine and Surgery, entitled "*Two Lectures on Water, Delivered to his Class, at Lebanon, Tennessee, by F. H. Gordon, M. D.*," we select the following extracts.—EDITOR BUFFALO MEDICAL JOURNAL.

Fomentation is a mode of applying warm water, by dipping flannel cloths in it, and applying them to the surface. It is an old remedy, and is much used at this day. But it is the least efficient of all the modes, because there is but little force in the application, and the cloths cool so rapidly as to require frequent changing Yet from its convenience, it answers a valuable purpose in many instances, where a more powerful effect is not demanded.

The poultice is a much more potent antiphlogistic, and in subduing local inflammation it is deservedly popular. Yet there are frequent failures in securing the good effects of this remedy, because of the injudicious manner of its application. The poultice, to do great service, must be *large, light, and frequently changed.* It must be large, because a small poultice cools too speedily; it must be light, because otherwise its weight would oppress the patient; and it must be frequently changed, to keep up a temperature as nearly uniform as possible. To accomplish all of these objects, wheat bran is the best material for a poultice, and should be managed in the following manner: Suppose you have a case of peritonitis, put ten quarts of wheat bran in an iron pot; gradually add water, and stir till the whole mass is *merely moist* or damp throughout. Now heat it, and stir occasionally till it is quite hot. Put half of this in a bag not less than eighteen inches square, (a pillow case is a suitable bag for this purpose,) spread the bran out, and apply the poultice, as hot as the patient can bear it, to the surface of the abdomen. At the end of twenty minutes have the remainder of the bran ready in another bag, and apply it the instant the former poultice is removed. Return the cool bran of the former poultice to the pot, to be ready to be reapplied at the end of twenty minutes more; and thus continue to change the poultices every twenty minutes, so as to keep up a temperature as nearly uniform as possible. Poulticing in this way for four or five hours will do more in subduing inflammation than a little and badly managed one could in a week. In fact, a little poultice is the mere semblance of a remedy; but when all of the conditions here named are observed, the poultice is powerful. I have named wheat bran as the best material; but poultices are made of various substances, many of which are supposed to possess some peculiar virtues, apart from the water they contain. But I suppose that the remedial agency of all or nearly all poultices is due to the water alone, which they contain. The tansy, hot ashes, cedar leaves, yarrow, corn mush, and wheat bran are only means of absorbing warm water, and holding it in contact with the surface. And we frequently see poultices medicated with oak ooze, vinegar, and other means, under the belief that these means have some agency in reducing the inflammation. Such medications of course do no injury, and I never object to them, but gratify the friends of the patient by allowing any such medication, provided the poultices are made large and hot, and are changed frequently; for my reliance is on the water in the poultice. * *

The stream is the most valuable form of applying warm water *for the cure of local inflammation.* I have been familiar with it for years. It had been used by surgeons to relax the muscles in reducing luxations; but I am not aware of its having been employed for the cure of local inflammation before I introduced it into the practice, For this latter purpose I am acquainted with no remedy which can equal it in value. I do not wish to overrate it, or to appear to exaggerate its powers, for this would prevent a proper estimate from being placed upon it. Yet, to those who have not tried the remedy, the whole truth will look like exaggeration. Cups, leeches, blisters, and the lancet, do not equal it. The stream is a mode of applying warm water which complies with all of the conditions requisite to make it efficacious. It is *more constant,* may be *longer continued,* and applied with *more force* than any other application of water. The stream may be conducted almost without variation of temperature or force for hours, and, falling *only upon the suffering point,* it does not prostrate the patient like the bath, and hence may be continued long enough to greatly subdue the local inflammation before there is much depression of the vital powers. The *force* of the stream is an item of peculiar importance, because it is incomparably greater than that of any other mode of using warm water. It is a well known fact, that *fluids in motion* manifest their peculiar powers in a much higher degree than when at rest. The sultry air of a summer's day, which almost melts us by its quiescence, may, when put in motion, make hail, and chill the body. Electricity, which gently pervades our bodies at all times, and stimulates all of the vital movements without misrule, if disturbed in its equilibrium, and transmitted through the organs, with its incalculable velocity, will produce instant disorganization and death. Caloric, which pervades all of the tissues, and without the vital functions could not be performed for one moment, does no injury while latent or in gentle motion; but when a large amount passes in or out of any point of the surface in a short time, destruction is the instant result. In like manner, water at rest in contact with the body will abstract or impart caloric according to its temperature, in so gradual a manner as to cause very striking results; but when a swift current of water strikes the surface, its effects are manifestly in proportion to its velocity. Hence the stream of warm water as far surpasses in virtue the vapor bath and fomenting cloths, as the gentle breeze which wafts a feather is transcended by the tornado which uproots the sturdy oak.

But this, like all other remedies, may lose more than half of its virtues by being badly applied; it is, therefore, important that some special directions be given for its employment. Your object is to cause a *constant stream to fall with force upon the suffering point.* You may accomplish all of these objects by pouring with two pitchers, alternately, so as never to stop the stream longer than is necessary to exchange an empty pitcher for a full one. Or two coffee-pots or tea-pots will answer the same purpose. But the syphon is far the best instrument. A leaden tube from two and a half to three feet long, or a bent tin tube, or two joints of cane mitred together, secured with twine, and made air-tight, will answer the purpose. The outer leg of the syphon must be from two to four inches longer than the one placed in the water. The diameter of the instrument must be from one-eighth to three-eighths of an inch, according as the patient is prostrated or vigorous. To understand the application of this instrument, particularly on the trunk, suppose you wish to apply the stream in a case of peritonitis. Take every thing from the bedstead down to the cords; fold the bed-clothes, and place them on the cords so as to make two beds of them, of equal height, with an interval of two inches between them, about where the umbilicus of the patient will be when he is placed upon them. Now place the patient, supine, upon the beds, with his umbilicus over the interval, and a pillow under his head. Two blankets must cover the patient above and below the umbilicus, so as to leave a space of four inches between them. Pass two silk handkerchiefs around the body of the patient, one just above and the other just below the umbilicus, with their ends hanging through the cords to conduct the water. Have the clothes of the patient well pulled up, so that they may not get wet. A tub is placed under the bed to catch the water. A plank rests on the head and foot board, on which a bucket of warm water is placed. One end of the syphon is put into the bucket and made steady by a weight tied to it, and the other end presents over the umbilicus of the patient. By applying the mouth to the outer end and sucking, the stream begins, and ought to be adjusted to make it fall upon the most tender point. It should fall five or six inches, from the end of the syphon to the skin of the patient, or as far as it can without making a spray. The water should be tempered in a separate vessel, and poured in the one which feeds the syphon, so as to keep it full and cause the stream to be uniform in temperature and force. The stream should fall constantly in the same place, because its effects will thus be greater than if moved frequently. The length of time consumed in using the stream must vary from one to five hours, according to the strength of the patient. Mustard plasters on the hands and feet will enable him to stand it much longer than without them. The rule is to continue the stream till the patient's pulse becomes small and frequent; and the higher the temperature of the water the longer it can be tolerated without faltering of the pulse. What should be the temperature of the water? At the beginning it should be about ninety degrees, Fahrenheit, and gradually increased till it reddens the skin slightly; this will be between one hundred and one hundred and fifteen degrees, according to the excitability of the patient. It is usual for patients to enjoy the stream finely for the first hour or two, but afterwards they become restless and insist on moving. They must be carefnlly instructed never to move themselves, for their clothes and bed would get wet. By rolling up a towel and placing it under one shoulder, and another under the hip of the same side, the patient will be made to rest for a while, and when he again becomes tired, place the towels under the opposite hip and shoulder. In this way you may make him contented till the bathing is ended, and all means should be employed for this purpose, for the effects of the stream increases almost in fourfold proportion with the time. In fact the last hour appears to accumulate as much good as the three preceding ones.

The first effect of the stream is on the point where it falls, subduing the inflammation for a small space around. The reduction gradually extends to more remote parts, as may be proven by gentle pressure being gradually tolerated progressively outward from where the stream falls. And, in the case of peritonitis before us, the legs can be completely extended without pain.

The second effect is upon the muscular, circulatory, and nervous systems. General relaxation ensues; the pulse loses its hardness, becomes full and elastic, and ultimately, is small and frequent, retains its softness. At first, the patient is soothed; all pains cease and he falls to sleep, but afterward awakes and becomes restless and weary of his position. These constitutional effects are so gradual, that you may almost, and sometimes entirely, subdue the local inflammation, before the patient becomes so much depressed as to require the discontinuance of the remedy. This is the excellency of the stream—that it acts with power enough *upon the point of disease* to subdue the inflammation in part or entirely, with but temporary depression of the general energies. The general febrile symptoms moderate and disappear while the stream flows, with as much certainty as can be expected from the lancet, and yet without ultimate loss of strength. It thus often does away the necessity of bleeding, and may be used in cases of great prostration, where no one would think of using the lancet. To a considerable extent, the warm stream supercedes the painful practice of blistering, relieving the disease without it; and where it does not subdue the local lesion entirely, it always diminishes it greatly, if continued long enough, and being on the decline, the blister plaster may be at once applied, with full prospect of good effects. And to speak in general terms, in all cases of local inflammation, the patient gets well sooner by the aid of the stream, than he could possibly without it, and suffers incomparably less during his illness. Unlike bleeding, purging, blistering, and all other powerful means of subduing inflammation, the warm stream may be repeated as often as may be needed. After all that has been said, need it be urged that we have no remedy so potent in reducing local inflammation? Let it be well tried, in a variety of cases, and you will be astonished at its effects. To see so pleasant a remedy relieve the most intense agony, remove all soreness, subdue the fever, and restore the pulse almost to its normal characteristics, in a few hours, almost passes belief. And though you will not be fortunate enough to see this picture universally, yet you may often be gratified with the sight.

You ask, in what cases is the stream applicable? I reply, *in all cases where there is local inflammation*, no matter what may be the name of the disease, whether acute or chronic, the stream will do good, and the nearer it can be made to fall on the point of the most intense suffering the better. It is true, that its effects will be more striking in acute disease, but it will be serviceable in chronic also. In mild or slight cases, I do not hesitate to direct the stream upon the suffering point.

I have watched this remedy in its extensive application, for many years, and could adduce from memory and my notes, a very long list of cases, tending to show its value; but must be content to bring forward comparatively few of them, taken promiscuously from the mass in my possession, to which I ask your patient attention.

The cases are omitted.—EDITOR BUFFALO MEDICAL JOURNAL.

